# Identifying the Optimal Parameters to Express the Capacity–Activity Interrelationship of Community-Dwelling Older Adults Using Wearable Sensors

**DOI:** 10.3390/s22249648

**Published:** 2022-12-09

**Authors:** Emily Wright, Victoria Chester, Usha Kuruganti

**Affiliations:** Andrew and Marjorie McCain Human Performance Laboratory, Faculty of Kinesiology, University of New Brunswick, Fredericton, NB E3B 5A3, Canada

**Keywords:** accelerometer, exercise, mobility, physical functioning, quality of life

## Abstract

Mobility is the primary indicator of quality of life among older adults. Physical capacity (PC) and physical activity (PA) are two determinants of mobility; however, PC and PA are complex constructs represented by numerous parameters. This research sought to identify the optimal parameters that may be used to represent PC and PA of older adults, while exploring the interrelationship of these two constructs. Participants were 76 community-dwelling older adults (M age = 74.05 ± 5.15 yrs.). The McRoberts MoveTest was used to objectively measure PC in the laboratory with the following tests: the Short Physical Performance Battery, the Sway test, Sit to Stands, and the Timed Up and Go. PA was then measured at home for one week using the McRoberts USB Dynaport. Correlation analyses resulted in 55% and 65% reductions of PC and PA parameters, respectively. Clustering identified five representative PC parameters and five representative PA parameters. Canonical correlation analysis identified a non-significant correlation between the two sets of parameters. A novel approach was used to define PC and PA by systematically reducing these constructs into representative parameters that provide clinically relevant information, suggesting that they are an accurate representation of one’s PC and PA. A non-significant correlation between PC and PA suggests that there is no relationship between the two in this sample of community-dwelling older adults. The research provided insight into two important determinants of older adult mobility, and how they influence each other.

## 1. Introduction

The population of Canada is rapidly aging. Those 65 years of age and older account for 17.2 percent of the population, and this number is expected to rise to 25 percent by the year 2036 [1,2]. Mobility is the primary indicator of quality of life (QoL) among this demographic, and physical activity (PA) and physical capacity (PC) are two important determinants of mobility [3,4]. However, very little is known about the mobility levels of older Canadians. The benefits of PA are well-known, including reduced risk of cardiovascular disease and mortality [5,6], and reduced onset of mobility impairments [7]. PA may lessen the overall amount of time spent with mobility impairments by as much as 25 percent [8], a particularly important finding as patients indicate that total duration of impairment has a greater influence on QoL than initial occurrence alone [9]. 

PC is a measurement of one’s ability to carry out basic activities of daily living and is imperative to independence [10]. PC is influenced by several systems, including cardiopulmonary, vestibular, and muscular [11,12]. Unfortunately, decline in these systems due to the natural aging process, leads to decreased capacity. Capacity is an underlying factor in one’s mobility [13] and as such, lower capacity may lead to lower mobility. Research shows that mobility itself is a key indicator of QoL [3,4]; therefore, declines in mobility are often paralleled by declines in QoL. For example, Davis and colleagues [14] reported that scores on common capacity measures (e.g., Timed Up and Go (TUG)) can predict health related QoL (HRQoL) in men, and are correlated with women’s baseline HRQoL. This emphasizes the influential relationship capacity has on HRQoL, and therefore it must be considered when evaluating mobility.

Due to its influence on mobility, the interrelationship between one’s physical capacity (‘what can they do’) and their physical activity (‘what do they do’) has been studied. A linear relationship between PC and PA is apparent, with much research suggesting those who engage in high levels of PA also present with higher PC [15,16]. Additionally, many PC outcomes are significantly and positively correlated with PA outcomes [16]. The positive PC–PA correlation suggests that physical activity level may mediate the relationship between PC, mobility, and thus, QoL. However, studies to date are limited in their representations of PC and PA. PC is often represented only by Short Physical Performance Battery (SPPB) scores, 6 Minute Walk Test (6MWT) distances, and time taken to complete other performance measures (e.g., TUG; [15,16,17,18,19]). PA is typically represented by either daily moderate to vigorous physical activity (MVPA), or step count [15,17,19]. However, PC and PA are complex constructs represented by numerous parameters, and it is possible that more optimal representations exist and have been ignored. For example, although two participants may present with the same score on the SPPB, there may be individual differences in sway displacement or sit to stand (STS) power that may provide further discriminatory or classification information. Additionally, advancements in wearable technology allow numerous PC and PA parameters to be easily measured through sensors such as the McRoberts MoveTest and MoveMonitor [20]. Together, these devices provide hundreds of outcome parameters pertaining to mobility that may aid in the discovery of additional, optimal parameters that could best represent the PC and PA of community-dwelling older adults. 

This study aimed to provide a novel and comprehensive assessment of the relationship between PC and PA in community-dwelling older adults using wearable sensors. Physical capacity was measured once in the lab setting, while physical activity was assessed over a one-week period. Data reduction techniques were used to identify the optimal parameters to express the interrelationship between PC and PA of community-dwelling older adults, as measured by the McRoberts MoveTest and MoveMonitor wireless health sensors. 

## 2. Materials and Methods

### 2.1. Participants

A total of 105 community-dwelling older adults were recruited for this study. Of these 105 participants, 28 were eliminated due to incomplete data and 1 was eliminated due to drop-out, leaving a total sample of 76 (N = 76) participants (21 males and 55 females). Participants were required to have at least five valid days of wear-time and were excluded (as incomplete data) if they accumulated less than 75 percent wear-time on more than 2 days. Participant ages ranged from 65 to 90 years (M = 74.05, SD = 5.15). 

Additional participant characteristics are presented in Table 1. Recruitment was through word of mouth, advertisements on social media, and the placement of posters in physiotherapy clinics, fitness and community centers, churches, and the local university campus. Interested participants contacted the laboratory, via telephone and email, and received a full briefing of the study’s purpose before scheduling a testing time. Inclusion criteria included the ability to (1) adequately communicate in English, (2) stand and ambulate without walking devices, and (3) live independently (i.e., not in a care-home or receiving assistance). Exclusion criteria included (1) any mental or psychiatric illness prohibiting participation, (2) individuals with leg/foot amputations, and (3) anyone with a physical or neurological condition that resulted in atypical gait. Participants were screened for cognitive impairment and were required to receive a minimum score of three out of five on the Mini-Cog©. Participants also completed a Physical Activity Readiness Questionnaire for Everyone (PAR-Q+) to determine if physical activity was safe. No participants were excluded based on their responses. All participants completed, or had their third-party complete, an informed consent form prior to participation. The study was approved by the Research Ethics Board at the University of New Brunswick.

### 2.2. Instrumentation

All PC data were collected using the McRoberts MoveTest (MT 50089) [20] at a sampling frequency of 100 Hz. This sensor contains a tri-axial accelerometer, gyroscope, and magnetometer. The following tests were used to measure participants’ physical capacity: 6 Minute Walk Test (6MWT), the Short Physical Performance Battery (SPPB), a Sway Test, and the Timed Up and Go (TUG). A total of 112 physical capacity parameters were collected from the MoveTest. For more specific information regarding the type and scope of collected parameters, refer to McRoberts [20]. The tests were completed in the following order: 6MWT, Sway test, SPPB, and Timed Up and Go (TUG), with breaks provided as needed. 

### 2.2.1. 6MWT

The 6MWT assesses aerobic capacity and endurance. Participants were instructed to walk along a straight trajectory between two pilons ten meters apart. On both ends, participants completed a 180 degree turn and continued walking in the opposite direction, until reaching the opposite turning point. Participants walked in this continuous loop for six minutes straight, only stopping if necessary. Any stops were counted by the monitor and the timer continued through the stop. The objective of this test was to walk as far as possible in the six minutes. A total of 13 outcome measures were collected from this test. These outcome measures included total distance walked, step length and frequency, turn duration, and velocity.

#### 2.2.2. SPPB

The SPPB assesses gait speed, STS performance, and balance and provides a combined result of the three tasks. Scores range from 0 (worst performance) to 12 (best performance), and each subtest is graded out of four. Gait speed was assessed using a 4 m walk. The faster of two trials was scored. The STS protocol required participants to rise from a chair of standard height (46 cm), without using upper-extremity support, five times as fast as possible. The fastest STS cycle was used for assessment. Balance was assessed using side-by-side, semi-tandem, and tandem stances, each held for ten seconds (or the best of their ability). A total of 12 outcome measures were collected pertaining specifically to the SPPB scores, and an additional 28 parameters were collected specifically for the STS sub-test. The SPPB general parameters include total score as well as gait speed and chair stand duration. The specific measures of STS include STS duration and sub-duration, trunk angles (flexion and extension), power (mean, peak, and rate of development), and velocity (linear and angular).

#### 2.2.3. Sway Test 

Balance information, supplemental to that from the SPPB, was collected using a tandem stance and a single-leg stance. Participants selected their dominant leg. Each stance was held for ten seconds (or to the best of their ability). A total of 38 parameters were reported for the sway test. Three-dimensional acceleration and angular velocity were generated, from which numerous other variables were calculated, including velocity, displacement in anterior-posterior and medial-lateral directions, path, area, and sway angle. 

#### 2.2.4. TUG 

The TUG is a measure of mobility. This study used a modified version and scoring system, validated by Podsiadlo and Richardson [21]. Participants were required to stand up from a chair of standard height (46 cm), without upper-extremity support, walk five meters, complete a 180-degree turn, return to the chair, and sit down. A total of 21 outcome measures were reported from the TUG including duration, sub-durations (sit-stand, stand-sit, walk 1, walk 2, turn 1, and turn 2), angular velocity, and acceleration. 

#### 2.2.5. Physical Activity Data

The McRoberts USB Dynaports [20] were used to collect PA data of participants at a sampling frequency of 100 Hz during a one-week period outside of the laboratory setting. This sensor contains a tri-axial accelerometer. Sensors were pre-programmed and automatically began measurement at 12 AM (00:00) the day immediately following in-lab testing. The sensors automatically shut off after the seven-day measurement. A total of 193 PA parameters were collected by the MoveMonitor across the following categories: 14 from The American College of Sports Medicine (ACSM) recommendations, 16 from the Netherlands Norm Gezond Bewegen (Dutch Healthy Moving Norms) recommendations, 6 from METs/MET minutes, 12 from Active Energy Expenditure, 12 from Total Energy Expenditure, 12 from Movement Intensity, 12 from Physical Activity Ratios/Level, 12 from Periods, 12 from Vector Magnitude Unit (Count in newest export), 18 from Walking, 6 from Transitions, 6 from the PROactive Tool, BMR, and 44 from Duration (mean, median, max, and total). The following movement classifications are included: inactive, active, moving, lying, sitting, standing, shuffling, static, cycling, stair-walking, walking, total, wear-time, and non-wear time. For more specific information regarding the type and scope of collected parameters, please refer to McRoberts [20].

### 2.3. Testing Procedure

All PC data were collected in the Andrew and Marjorie McCain Human Performance Laboratory using the McRoberts MoveTest (MT 50089) [20]. Informed consent was obtained from all participants. PC was measured during a single lab session that was approximately 40 min in duration. Participants completed the cognitive screening, a physical activity health questionnaire, and a demographic form. Following this, height (cm) and weight (kg) were measured, and participants were fitted with the MoveTest sensor before proceeding with PC testing. Sensors were worn on an adjustable elastic band, secured with Velcro, over or under clothing, and centred at the lower-lumbar region of the spine. The following tests were used to measure participants’ physical capacity: 6 Minute Walk Test (6MWT), the Short Physical Performance Battery (SPPB), a Sway Test, and the Timed Up and Go (TUG). A total of 112 physical capacity parameters were collected from the MoveTest. For more specific information regarding the type and scope of collected parameters, refer to McRoberts [20]. 

The 6MWT assesses aerobic capacity and endurance. Participants were instructed to walk along a straight trajectory between two pilons 10 m apart. On both ends, participants completed a 180 degree turn and continued walking in the opposite direction, until reaching the opposite turning point. Participants walked in this continuous loop for six minutes straight, only stopping if necessary. Any stops were counted by the monitor and the timer continued through the stop. The objective of this test was to walk as far as possible in six minutes. A total of 13 outcome measures were collected from this test. The SPPB assesses gait speed, STS performance, and balance, and provides a combined result of the three tasks. Scores ranged from 0 (worst performance) to 12 (best performance), and each subtest was graded out of four. Gait speed was assessed using a 4-metre walk. The faster of the two trials was scored. STS protocol required participants to rise from a chair of standard height (46 cm), without using upper-extremity support, 5 times, as fast as possible. The fastest STS cycle was used for assessment. Balance was assessed using side-by-side, semi-tandem, and tandem stances, each held for 10 s (or the best of their ability). A total of 12 outcome measures were collected pertaining specifically to the SPPB scores, and an additional 28 parameters were collected specifically for the STS sub-test. Balance information, supplemental to that from the SPPB, was collected using a tandem stance and a single-leg stance. Participants could select their dominant leg. Each stance was held for ten seconds (or to the best of their ability). A total of 38 parameters were reported for the sway test. The TUG is a measure of mobility. This study used a modified version and scoring system, validated by Podsiadlo and Richardson [21]. Participants were required to stand up from a chair of standard height (46 cm), without upper-extremity support, walk 5 m, complete a 180-degree turn, return to the chair, and sit down. A total of 21 outcome measures were reported from the TUG.

Following the collection of PC data, participants were provided with McRoberts Dynaports, and instructed on how to use and wear the devices. PA was measured for one week using the McRoberts Dynaports during regular daily activity. Sensors were worn on an adjustable elastic band, secured with Velcro, over or under clothing, centred at the lower-lumbar region of the spine. Sensors were pre-programmed and automatically began measurement at 12 AM (00:00) in the evening, following in-lab testing. The sensors automatically shut off after the seven-day measurement. Participants were instructed to go about their daily routine as usual to produce a baseline measurement of activity. The sensor was worn 24 h/day, seven days/week, excluding during water-based activities. At the end of the seven-day period, participants returned the sensors to the researcher and completed a post-test questionnaire regarding their activities that week. The monitors reported non-wear time (in percent of day) as determined by the manufacturer’s wear detection algorithm, based on a threshold of signal power. 

### 2.4. Data Analysis Procedure 

All analyses were completed using the statistical programs SAS, version 9.4m4 [22], and SPPS, version 25 [23] with an alpha level of 0.05. All data recorded by the MoveTest and MoveMonitor sensors were submitted online and processed by McRoberts software. Data were released in comma separated value files upon request to McRoberts [20]. 

#### 2.4.1. Data Reduction

First, capacity and activity parameters underwent independent reductions. This involved eliminating redundant parameters in which identical information was provided under another category. Parameters presenting with no variability (i.e., identical values across all participants) were also removed from consideration. All parameters not pertaining to an individual’s PA or sedentary time were also removed (e.g., “periods of non-wear time”). Correlation matrices were then generated within each parameter category, and between categories where high correlations were anticipated (e.g., total and active energy expenditure). Parameters with strong positive or negative correlations (0.80 or higher) were eliminated. This value was chosen as 0.80, which is the most typical cut-off used to indicate multi-collinearity between parameters [24,25]. 

#### 2.4.2. Cluster Analysis

Following data reduction, cluster analysis was used to further reduce parameters. Data were scaled to standardize the variable ranges. A hierarchical clustering model was then independently applied to the capacity and activity parameters. The Elbow Method was used to determine the optimal number of clusters (k = 5 per set, total k = 10), selected based on percentage of variance explained, where k identifies the number of clusters where any more would not give a better model [26]. From each cluster, the parameter with the lowest 1-R^2^ ratio was selected as a representative variable. Means and standard deviations were then generated for all ten representative parameters. 

#### 2.4.3. Canonical Correlation Analysis

To examine the relationship between capacity and activity, Canonical Correlation Analysis (CCA) was completed using the ten representative parameters. Data were again scaled to standardize the parameters’ ranges. Next, canonical correlation was completed, and the following were generated: canonical correlations, R^2^ values, eigen values, the approximate F values, and their corresponding significance (Wilks test), estimated coefficients for the capacity parameters, and estimated coefficients for the activity parameters. 

## 3. Results

### 3.1. Data Reduction 

A large data reduction, facilitated by correlation matrices and the removal of both redundant parameters and those with low variability, was completed for each of the physical capacity and activity parameters lists. A total of 193 of PA parameters were collected by the MoveMonitor. Data reduction resulted in a 65% decrease in the number of parameters. This left 69 PA parameters to be used for clustering, while 124 were excluded from further analyses. A total of 112 PC parameters were reported by the MoveTest. Following data reduction, 57 parameters remained to be used for clustering, representing a 55% decrease in parameters. Fifty-five (N = 55) parameters were excluded from additional analyses. Five (N = 5) of these parameters were removed on a conditional basis as they did not provide valuable information for this sample, but should be revisited in different populations (e.g., frail populations). 

### 3.2. Clustering

Hierarchical clustering and the elbow method were used to cluster the activity and capacity parameter lists and determine representative parameters.

#### 3.2.1. Physical Activity 

The Elbow Method recommended five clusters for the PA data. These five clusters explained 61% of variance. From these five clusters, the following representative parameters were selected (additional cluster characteristics are presented in Table 2):

- Active duration: the total duration (minutes) of standing, shuffling, cycling, and walking combined;
- Movement intensity: the average movement intensity (m/s^2^) of active time;
- Lying-Standing: the number of transitions from lying to standing;
- Walking duration ≥ 20 s: the total (i.e., cumulative) duration (minutes) of walking periods greater than 20 s;
- Inactive periods: the number of sitting and lying periods combined.

#### 3.2.2. Physical Capacity

The Elbow Method recommended five clusters for the PC data. These five clusters explained 47% of variance. From these five clusters, the following representative parameters were selected (additional cluster characteristics can be found in Table 3):

- STS duration: time (in seconds) taken to complete one complete sit-to-stand cycle (sit-to-stand, stand, stand-to-sit, sit) at the participants’ fastest pace;
- Displacement: the mean (in mm) of the absolute AP and ML displacement during sway;
- 6MWT Distance: the total distance walking (m) during the 6MWT;
- STS power: the mean power (watts) of the sit to stand transition;
- StandToSitflex: the total flexion range (in degrees) of the trunk during the stand to sit transition.

### 3.3. Canonical Correlation Analysis (CCA)

**Figure 1:**
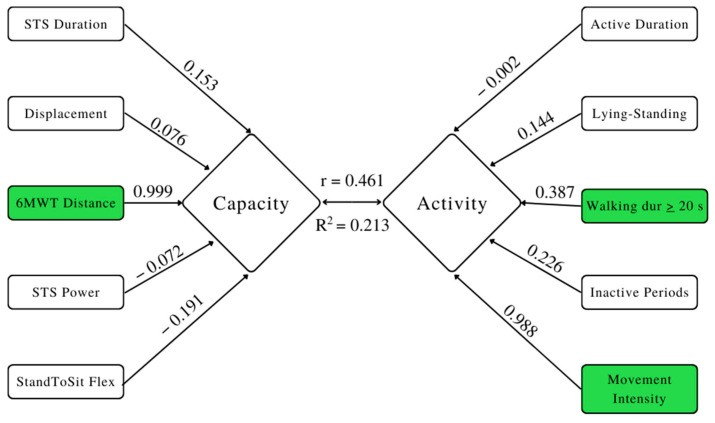
Model depicting the first canonical correlation between capacity and activity showing both the canonical correlation (r) and the amount of variability accounted for by the model (R^2^). The coefficients/weights for each variable are presented adjacent to their arrow. The significant contributors to the activity-capacity relationship are highlighted.

Canonical correlations were generated to explore the relationship between PC and PA, as defined by the representative parameters selected from the clustering models. The first (i.e., largest) correlation was 0.4611 and represented the strongest relationship and accounted for the largest amount of variability in the relationship. It has been depicted in a model for visual representation (Figure 1). The model presents the correlation (r = 0.4611), the variability accounted for (r^2^ = 0.213), as well as the weights for each of the PC and PA parameters. The correlation was non-significant (*p* = 0.157). The significant contributors to the activity–capacity relationship were the 6MWT distance, walking duration (≥20 s), and movement intensity.

## 4. Discussion

The purpose of this research was to comprehensively assess and identify the optimal parameters to express the PC-PA relationship of community-dwelling older adults. A large data reduction was completed, aided by correlation matrices and clustering, which identified five PC and five PA parameters (Figure 1). PC was represented by activity duration, movement intensity, lying to standing transitions, walking duration (≥20 s), and inactive periods. PA was represented by STS duration, STS power, StandToSit flex (trunk flexion range), 6MWT distance, and displacement (mean of the absolute AP and ML displacement during sway). Using these parameters, CCA was used to investigate the relationship between PC and PA, and a non-significant correlation was found. 

Clustering served to identify the optimal parameters to represent PC and PA of community-dwelling older adults. Many of the selected PC parameters are comparable to those reported in the literature. For example, 6MWT distance has been correlated with both community mobility and mortality [27,28]. Although less commonly reported in the literature, the other identified variables also provide valuable clinical information. For example, fall risk also relates to centre of pressure balance measures such as displacement [29]. In fact, research indicated that sway, particularly in the mediolateral direction, was the single best predictor of future falling [30]. Additionally, Van Lummel et al. [31] reported that both longer STS duration and greater trunk flexion range have been associated with weaker subjects (i.e., lower hand-grip strength), while STS power is considered a marker of frailty, and more important than strength in maintaining functional capacity [32]. 

PA clustering identified unique parameters that provide clinical value, but their comparability is limited as many of them are rarely reported. For example, movement intensity of active time (m/s^2^) is not often reported in these units; however, its values are the precursors to METs, and in both cases, higher values indicate higher intensities. The health benefits of engaging in high intensity PA have been repeatedly supported throughout the literature [33,34]. Walking duration, accumulated in bouts ≥20 s, was highlighted as compared to shorter bouts (10–19.99 s). Research indicates that when total walking time remains consistent, walking totaled in fewer, longer bouts leads to improved health benefits compared to many shorter ones [35,36]. While these studies often compare bouts of thirty minutes to three ten-minute bouts, it is possible that the results may be extrapolated to similar ratios. 

Recent research has also investigated the impact of not only total duration of sedentary behaviours, but the pattern in which they are accumulated [37,38]. For example, Diaz et al. [37] found that when total sitting duration remained the same, participants who sat for bouts fewer than 30 min (and thus had a greater number of sitting periods) had the lowest risk of death compared to those who continuously sat for 60 or 90 min bouts. Therefore, the number of inactive periods per day may provide valuable mortality information when combined with total duration. Total duration of active time is total time spent engaging in non-sedentary activity of any intensity. While this definition is not often used in the literature, it emphasizes the importance of movement, as opposed to sedentary behavior, a statement that has been supported by past research [39,40]. Lying to standing transitions are also not reported within the literature. However, future work should consider this transition as it leads to a rapid decrease in blood pressure, or orthostatic hypertension (OH) [41]. OH, is prevalent in older adults and is associated with both falls, and impaired physical performance [41]. In other words, lying to standing transitions may lead to important, fall-predictive information. 

CCA was completed to examine the relationship between PC and PA. The largest canonical correlation was a positive, moderate 0.4116, similar to the values reported by Van Lummel and colleagues [16] where PC had moderate correlations (r = −0.29 to 0.68) with various measures of PA (e.g., mean duration of locomotion). Similarly, other studies have reported strong and positive associations between PA and PC [15,16,19]. However, in the present study, Wilks test identified this as a non-significant correlation, meaning there was no relationship between PC and PA. There are many possible reasons for this result. For example, high PC does not always result in high PA due to a variety of confounding factors (e.g., low motivation, busy schedule, lack of interest, acute injury). Similarly, low PC does not always reflect low PA as one may be committed to a regular exercise regimen that is reflective of their current PC (e.g., walking instead of running, or bodyweight exercises instead of heavy lifting). This study highlights the complex nature of the PC–PA relationship and the variety of mediating variables that must be considered. 

Previous research examining the PC–PA relationship has utilized a limited number of parameters to enhance consistency and comparability. For example, several studies rely on the 6MWT distance [15,18,42] as a representation of PC. The present study identified this parameter to be the primary PC contributor to the PC–PA relationship. While further work is required to determine if these results are replicable, the current findings appear consistent with previous research. Similarly, step count and PA are often synonymously used, with many studies reporting increases in step count as increases in PA [17,43,44]. Although step count is inversely related to several health conditions (e.g., hypertension and metabolic disease [44,45]), it provides only volume-based information (as opposed to frequency or duration) and is not comparable to current PA guidelines [46]. The results of the present research provide support for the continued measurement of step count. While step count was not identified from the clustering model it was highly correlated (r = 0.92), with walking duration in bouts ≥20 s being identified as a representative PA parameter. However, movement intensity of active time was the most significant parameter (Figure 1). This suggests that studies considering only step count as their representation of PA would be enhanced with the addition of an intensity measure. 

While the non-significant results of the CCA may appear contradictory to previous research, this study defined PC and PA using a novel approach. Additionally, this study provided a baseline representation of the PC–PA relationship in well-functioning older adults. As individuals with walking aids and atypical gait patterns were excluded, those with a mobility impairment were likely also excluded, meaning participants presented with what may be subjectively labelled as ‘good capacity’. High PC is unlikely to limit one’s PA; as such, the PC–PA relationship may be mitigated. Future work should investigate these parameters in clinical populations, such as those with COPD or Parkinson’s Disease, as we expect the relationship would change. 

To our knowledge, this is the most comprehensive assessment of older adult PC and PA using wireless monitoring. Although an effort was made to be all-inclusive, it is possible that relevant parameters were still excluded. For example, this study did not have any measure of upper-extremity strength, such as hand-grip strength. Additionally, there are many techniques that may be used to determine the number of clusters, and if alternative methods were used, it is possible that different parameters would be highlighted. Future work should investigate if the parameters deemed important in this research, for this population, are consistent when alternative methods are used, and/or additional parameters are included. It should also be noted that the impact of sex on the PC–PA relationship was not considered in this study, due to the unequal ratio of males to females in the present sample. It is recommended that this factor be explored in future studies.

## 5. Conclusions

Overall, this study provides important work that aids in the understanding of the physical capacity–activity relationship. While previous research has investigated the PC-PA relationship, this study used a novel approach of systematically reducing two complex constructs into two smaller sets of optimal parameters. This ensures that parameters that provide important classifying information are not neglected based on precedence. Moreover, exploring the relationship between these parameters in a baseline population allows for future comparisons with clinical populations. The results of this study serve to highlight important parameters in the PC–PA relationship, and how the two are related to each other. Participants benefited from an increased understanding of their personal capacity levels, and the value of physical activity. Participants were also given access to their physical activity records. This information serves to increase self-awareness on an individual level, as well as provide baseline data for the older adult population as a whole. With the older adult population of Canada rapidly growing, it is crucial to have a better understanding of PC–PA levels, and the relationship between the two, as this will lead to an improved understanding of older adult mobility. This knowledge will lead to more appropriate interventions for mobility impairments and low activity levels that will serve to improve the health, and quality of life of participants.

## Figures and Tables

**Table 1:** Demographic characteristics for the sample (N = 76).

	Age (Years)	Weight (kg)	Height (cm)
Men (N = 21)	73.29 ± 5.12	85.38 ± 21.89	172.38 ± 5.96
Women (N = 55)	74.35 ± 5.18	66.02 ± 10.16	159.87 ± 7.25
Total (N = 76)	74.05 ± 5.15	71.37 ± 16.58	163.33 ± 8.85

Note. Values are presented as mean ± SD. Weight is measured in kilograms (kg), and height is measured in centimeters (cm).

**Table 2:** Clustering characteristics for the physical activity parameters selected.

5 Clusters		R^2^ with:		
Cluster	Variable	Own Cluster	Next Closest	1-R^2^ Ratio
1	Active duration	0.8430	0.3815	0.2538
2	Movement intensity	0.9792	0.0155	0.0211
3	Lying-Standing	0.6973	0.0546	0.3202
4	Walking duration ≥ 20 s	0.8775	0.2030	0.0607
5	Inactive periods	0.8837	0.0740	0.1255

**Table 3:** Clustering characteristics for the physical capacity parameters selected.

5 Clusters		R-Squared with		
Cluster	Variable	Own Cluster	Next Closest	1-R^2^ Ratio
1	STS duration	0.9354	0.0868	0.0707
2	Displacement	0.8770	0.0362	0.1276
3	6MWT distance	0.7350	0.0463	0.2778
4	STS power	0.8140	0.0366	0.1931
5	STS flex	0.7452	0.1572	0.3024

## Data Availability

Data are not available, see above.
